# Ultrafast Terahertz Field Control of the Emergent Magnetic and Electronic Interactions at Oxide Interfaces

**DOI:** 10.1002/adma.202512328

**Published:** 2025-11-24

**Authors:** Abigail M. Derrico, Martina Basini, Vivek Unikandanunni, Jay R. Paudel, Mikhail Kareev, Michael Terilli, Tsung‐Chi Wu, Afnan Alostaz, Christoph Klewe, Padraic Shafer, Andrei Gloskovskii, Christoph Schlueter, Claus M. Schneider, Jak Chakhalian, Stefano Bonetti, Alexander X. Gray

**Affiliations:** ^1^ Department of Physics Temple University Philadelphia PA 19122 USA; ^2^ Department of Physics University of California Berkeley CA 94720 USA; ^3^ Department of Physics Stockholm University Stockholm 10691 Sweden; ^4^ Physics Department ETH Zurich Zürich 8093 Switzerland; ^5^ Institute of Applied Physics University of Bern Bern 3012 Switzerland; ^6^ Department of Physics and Astronomy Rutgers University Piscataway NJ 08854 USA; ^7^ Peter Grünberg Institut (PGI‐6) Forschungszentrum Jülich GmbH D‐52425 Jülich Germany; ^8^ Advanced Light Source Lawrence Berkeley National Laboratory Berkeley CA 94720 USA; ^9^ Deutsches Elektronen‐Synchrotron DESY 22607 Hamburg Germany; ^10^ Department of Molecular Sciences and Nanosystems Ca’ Foscari University of Venice Venice 30172 Italy

**Keywords:** complex oxide heterostructures, interfacial ferromagnetism, ultrafast dynamics, X‐ray spectroscopy and scattering

## Abstract

Ultrafast electric‐field control of emergent electronic and magnetic states at oxide interfaces offers exciting prospects for the development of the next generation of energy‐efficient devices. Here, it is demonstrated that the electronic structure and emergent ferromagnetic interfacial state in epitaxial LaNiO_3_/CaMnO_3_ superlattices can be effectively controlled using intense, single‐cycle THz electric‐field pulses. A suite of advanced X‐ray spectroscopic techniques is employed to measure a detailed magneto‐optical profile and the thickness of the ferromagnetic interfacial layer. Then, a combination of time‐resolved and temperature‐dependent optical measurements is used to disentangle several correlated electronic and magnetic processes driven by ultrafast, high‐field THz pulses. Sub‐picosecond non‐equilibrium Joule heating of the electronic system is observed, ultrafast demagnetization of the ferromagnetic interfacial layer, and slower dynamics indicative of a change in the magnetic state of the superlattice due to the transfer of spin‐angular momentum to the lattice. These findings suggest a promising avenue for the efficient control of 2D ferromagnetic states at oxide interfaces using ultrafast electric‐field pulses.

## Graphical abstract

Intense single‐cycle THz electric‐field pulses enable ultrafast control of emergent electronic and magnetic states at LaNiO_3_/CaMnO_3_ interfaces. Combined X‐ray and optical probes reveal sub‐picosecond demagnetization driven by spin‐polarized currents and slower magnetoelastic dynamics linked to spin–lattice coupling. These intertwined processes uncover a pathway to manipulate interfacial ferromagnetism in correlated oxides using ultrafast THz‐field control.

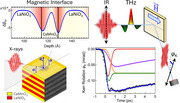

## 1 Introduction

The study and design of complex‐oxide heterostructures that exhibit emergent electronic and magnetic phenomena at their interfaces has become an active area of research in condensed matter physics and materials science.^[^ ^1^, ^2^, ^3^, ^4^, ^5^, ^6^, ^7^ ^]^ Particularly intriguing are material systems where the intricate interplay among different degrees of freedom at the interface leads to unique functional properties and states.^[^ ^8^, ^9^, ^10^, ^11^, ^12^, ^13^, ^14^, ^15^ ^]^ Ultrafast electric‐field control of these emergent electronic and magnetic states offers the potential for developing new types of atomically‐thin spintronic devices that support transient states, require minimal energy consumption, and operate at speed limits governed only by the fundamental laws of physics.^[^ ^16^, ^17^, ^18^, ^19^, ^20^ ^]^

The burgeoning field of ultrafast terahertz (THz) science has been exploring this promising avenue using both resonant and non‐resonant THz electric‐field pulses to understand and control the ultrafast processes and dynamics of charge carriers, spins, and phonons in a wide variety of material systems.^[^ ^20^, ^21^, ^22^, ^23^, ^24^, ^25^, ^26^, ^27^, ^28^, ^29^, ^30^, ^31^, ^32^, ^33^, ^34^, ^35^, ^36^, ^37^, ^38^, ^39^, ^40^ ^]^ Notably, several recent studies have showcased the effectiveness of THz excitation in triggering and controlling seminal physical phenomena in oxides, including insulator‐metal transition,^[^ ^20^, ^21^ ^]^ ferroelectricity,^[^ ^22^ ^]^ and transient superconductivity.^[^ ^23^, ^24^ ^]^ However, investigations of THz interactions with magnetically ordered materials have mainly focused on the dynamics and coherent spin control in bulk single‐crystal, bulk‐like thin‐film insulating antiferromagnets,^[^ ^25^, ^26^, ^27^, ^28^, ^29^, ^30^, ^31^, ^32^, ^33^, ^34^, ^35^ ^]^ and metallic ferromagnets,^[^ ^36^, ^37^, ^38^, ^39^, ^40^ ^]^ but not in epitaxially engineered interfaces hosting strong correlations between electronic and magnetic degrees of freedom.

Here, we consider ultrafast THz control of the emergent ferromagnetic order induced by interface engineering in an archetypal strongly correlated heterostructured oxide superlattice consisting of antiferromagnetic CaMnO_3_ and paramagnetic LaNiO_3_.^[^ ^41^, ^42^, ^43^, ^44^, ^45^ ^]^ The use of intense single‐cycle THz electric‐field pulses is designed to effectively simulate the action of an ultrafast picosecond current pulse, which has been recently shown to induce magnetization switching in a metallic ferromagnetic heterostructure.^[^ ^46^ ^]^ We demonstrate that the electronic structure and emergent interfacial ferromagnetic state at the interface can be effectively controlled using such ultrafast THz electric‐field pulses.

For our study, we utilize a combination of polarization‐dependent X‐ray absorption spectroscopy with magnetic circular dichroism (XAS/XMCD) and soft X‐ray resonant magnetic reflectivity (XRMR)^[^ ^47^ ^]^ to measure the detailed magneto‐optical profile of the LaNiO_3_/CaMnO_3_ interface and determine the characteristic thickness of the ferromagnetic interfacial layer. We then employ a combination of temperature dependent time‐resolved magneto‐optical Kerr effect (tr‐MOKE), optical reflectivity, and transmissivity measurements to disentangle several interrelated electronic and magnetic processes driven by ultrafast high‐field (≈1 MV cm^−1^) THz electric‐field pulses and evolving with different characteristic timescales.

Our measurements reveal several distinct ultrafast responses to THz excitation. During the first picosecond, an electronic response marked by non‐equilibrium Joule heating due to electron‐electron scattering occurs simultaneously with the THz pulse.^[^ ^48^ ^]^ This is accompanied by an ultrafast increase in the metallicity of the superlattice, which, in equilibrium, consists of metallic LaNiO_3_ and insulating CaMnO_3_.^[^ ^20^, ^49^, ^50^ ^]^ At temperatures below *T*_C_ (≈80 K), these sub‐ps electronic dynamics become strongly modulated by a concomitant magnetic response indicative of rapid (≈270 fs) demagnetization of the ferromagnetic interfacial layer driven by THz‐field‐induced nonequilibrium spin‐polarized currents.^[^ ^37^, ^51^, ^52^, ^53^ ^]^ Subsequently, the system experiences electron‐phonon thermalization. Thus, in addition to the initial sub‐picosecond response, slower, highly temperature‐dependent magneto‐optical dynamics emerge on a multi‐picosecond timescale, possibly signifying a change in the magnetic state of the superlattice due to the transfer of spin angular momentum to the lattice.

Our findings show that the several interrelated non‐equilibrium electronic and magnetic processes originating at the LaNiO_3_/CaMnO_3_ interface can be disentangled in the time domain. Additionally, a correlation between the charge and spin dynamics exists in this material system, and the electronic effects observed below *T*_C_ (≈80 K) are strongly modulated by the interfacial magnetism. This connection suggests a pathway for efficiently switching the 2D ferromagnetic states at oxide interfaces using ultrafast THz electric fields.

## 2 Element‐Specific and Depth‐Resolved Profiling of the Interfacial Ferromagnetism

In epitaxial LaNiO_3_/CaMnO_3_ superlattices, strongly correlated physics and competing ferromagnetic exchange interactions are intertwined at the interface, giving rise to quasi‐2D ferromagnetism.^[^ ^41^ ^]^ The emergent ferromagnetic order arises due to the interfacial charge transfer between LaNiO_3_ and CaMnO_3_^[^ ^45^ ^]^ and can be tuned by modulating the conductivity of LaNiO_3_, which undergoes a metal‐insulator transition in the ultrathin (few‐unit‐cell) limit.^[^ ^54^ ^]^ Thus, in superlattices with an above‐critical LaNiO_3_ thickness (*d* ≥ ≈4 u.c.), ferromagnetism mediated by double‐exchange interaction is stabilized on the interfacial Mn sites.^[^ ^41^ ^]^ Conversely, in the superlattices with a below‐critical LaNiO_3_ thickness (*d* < ≈4 u.c.) the charge transfer is suppressed and, therefore, only a very weak residual ferromagnetic signal is detected in some samples^[^ ^42^ ^]^ due to defect‐mediated phenomena.

For this study, a LaNiO_3_/CaMnO_3_ superlattice with ten repetitions containing bulk‐like metallic LaNiO_3_ layers with a thickness of eight unit cells (8 u.c.) and 4 u.c. of insulating CaMnO_3_ was synthesized on a single‐crystalline LaAlO_3_ (001) substrate using pulsed laser interval deposition.^[^ ^55^ ^]^ Epitaxial growth was monitored in situ via reflection high‐energy electron diffraction (RHEED). An additional control sample comprised the same number of repetitions (10) and the same CaMnO_3_ layer thickness (4 u.c.), but with ultrathin (2 u.c.‐thick) insulating LaNiO_3_ layers, was synthesized in the same batch. For brevity, we will refer to these two superlattices as 8LNO/4CMO and 2LNO/4CMO, respectively, indicating the LaNiO_3_ thickness. This pair of samples—one exhibiting interfacial ferromagnetism (8LNO/4CMO) and the other serving as a non‐magnetic control (2LNO/4CMO)—has been extensively studied alongside a wide range of LaNiO_3_/CaMnO_3_ superlattices with varying layer thicknesses and stacking sequences,^[^ ^41^, ^42^, ^43^, ^44^, ^45^ ^]^ all of which consistently demonstrate that the emergent magnetic behavior originates at the interfaces between the two oxides.

The structural, chemical, and magnetic properties of the superlattices are comprehensively characterized in Figures S1–S4 of the Supporting Information. Figure S1 (Supporting Information) shows high‐resolution X‐ray diffraction (XRD) data confirming the high crystalline quality and well‐defined superlattice periodicity of both the 2LNO/4CMO and 8LNO/4CMO samples. Figure S2 (Supporting Information) presents resonant and non‐resonant soft X‐ray reflectivity (SXR) spectra and model fits, demonstrating atomically sharp interfaces with interdiffusion limited to approximately one unit cell. Figure S3 (Supporting Information) displays synchrotron‐based hard X‐ray photoelectron spectroscopy (HAXPES)^[^ ^56^ ^]^ survey and valence‐band spectra that verify the expected chemical composition and reveal the thickness‐dependent suppression of the near‐Fermi‐level Ni 3*d* states associated with the metal‐insulator transition in LaNiO_3_. Finally, Figure S4 (Supporting Information) shows temperature‐dependent superconducting quantum interference device (SQUID) magnetometry data confirming the emergence of interfacial ferromagnetism in the 8LNO/4CMO superlattice, with a magnetic moment consistent with prior reports. Together, these results substantiate the structural integrity, interface quality, and interfacial magnetic behavior central to our conclusions.

In order to unequivocally establish the interfacial origin of the ferromagnetic signal detected in the 8LNO/4CMO superlattice by SQUID magnetometry (see Figure S4, Supporting Information), we carried out element‐specific and depth‐resolved magnetic characterization and profiling of the sample using XAS/XMCD and XRMR at the high‐resolution (100 meV) Magnetic Spectroscopy beamline 4.0.2 at the Advanced Light Source.^[^ ^57^ ^]^

**Figure 1:**
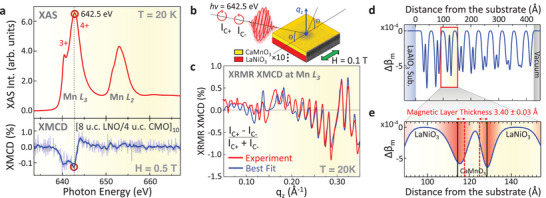
Element‐specific and depth‐resolved profiling of the interfacial ferromagnetism. a) Top panel: Bulk‐sensitive Mn *L*_2,3_‐edge XAS spectrum, measured in luminescence detection mode at *T* = 20 K and probing the entire depth of the 8LNO/4CMO superlattice, reveals a mixed (3+/4+) Mn valence state in the CaMnO_3_ layers. Bottom panel: XMCD spectrum measured in an in‐plane magnetic field of 0.5 T exhibits a significant magnetic signal of up to −0.15% at the Mn *L*_3_ edge (642.5 eV). The light‐blue spectrum represents raw data while the solid blue curve has been smoothed using the Savitzky‐Golay method. b) Schematic diagram of the XRMR‐XMCD measurements in the specular reflection geometry. c) XRMR‐XMCD asymmetry and the best fits to the experimental data measured at the resonant photon energy of the Mn *L*_3_ edge. Self‐consistent fitting of the data yields a detailed magneto‐optical profile of the superlattice, as shown in panels (d,e). d) Depth‐resolved magneto‐optical profile of the entire superlattice given by the modulation of the magnetic dichroism of the X‐ray optical constant Δβ_m_. e) Detailed magneto‐optical profile of the CaMnO_3_ layer and two adjacent LaNiO_3_ layers in the near‐central region of the superlattice. Typical thickness of the interfacial ferromagnetic layer is 3.40 ± 0.03 Å with a 2.39 ± 1.49 Å Névot‐Croce‐type interdiffusion present on either side.

The XAS spectrum of the Mn *L*_2,3_ absorption edges shown in the top panel of **Figure** **1**a exhibits excellent agreement with prior studies.^[^ ^44^, ^45^ ^]^ The spectrum reveals a low‐energy (640.5 eV) spectral feature attributed to the presence of Mn^3+^ cations, suggesting a mixed (3+/4+) Mn valence state in CaMnO_3_. This mixed valence state is required for the establishment of the ferromagnetic double exchange interaction.^[^ ^41^, ^42^, ^43^, ^44^, ^45^ ^]^

The resulting ferromagnetic state of Mn is then observed in the XMCD spectrum shown in the bottom panel. Due to the depth‐averaging nature of the XAS/XMCD technique, the XMCD signal originating from the ultrathin buried interfacial regions is weak (≈0.1%) compared to that of typical manganite films showing mostly depth‐uniform magnetization.^[^ ^58^ ^]^ However, this highly localized magnetic signal can be amplified by carrying out depth‐resolved *q*_z_‐dependent XRMR spectroscopy, which, in conjunction with X‐ray optical modeling, can be used to derive the detailed magneto‐optical profile of the superlattice.^[^ ^47^, ^59^ ^]^

The above‐mentioned XRMR measurements were carried out in the specular reflection geometry depicted schematically in Figure 1b. A resonant photon energy of 642.5 eV, corresponding to the maximum of the XMCD signal at the Mn *L*_3_ absorption edge, was used. The percent magnetic asymmetry (I_C+_ − I_C‐_)/(I_C+_ + I_C‐_) was recorded as a function of momentum transfer *q*_z_. The measurements were carried out in an applied in‐plane magnetic field of 0.1 T and at a sample temperature of 20 K, which is well below the reported T_C_ for this system (≈80 K). An additional XRMR spectrum at the photon energy corresponding to non‐resonant excitation (620 eV) was also recorded and analyzed (see Figure S2 in the Supporting Information).

The resulting experimental XRMR‐XMCD versus *q*_z_ spectrum is depicted as a red curve in Figure 1c. It is crucial to note that the measured *q*_z_ range spans both the first‐ and second‐order Bragg conditions (at ≈0.15 and ≈0.30 Å^−1^, respectively). Consequently, it encompasses detailed depth‐resolved information concerning the interfacial magnetic structure of the sample.^[^ ^59^, ^60^ ^]^

The XRMR–XMCD spectrum, together with the corresponding non‐resonant and resonant X‐ray reflectivity spectra shown in Figure S2 of the Supporting Information, was self‐consistently modeled using the X‐ray reflectivity analysis program ReMagX^[^ ^59^ ^]^ in accordance with the hierarchical three‐step fitting procedure described in Section 3 of the Supporting Information. The best theoretical fit is represented by the blue‐colored spectrum in Figure 1c, demonstrating a good agreement with the experimental data in terms of feature amplitudes, relative phases, and lineshapes. It is worth noting that the utilization of *q*_z_‐dependent (I_C+_ − I_C‐_)/(I_C+_ + I_C‐_) spectra considerably enhances the sensitivity of the fitting process. These spectra often reveal intricate lineshapes and numerous sharp modulations with varying amplitudes and shapes across the entire *q*_z_ range, thereby severely constraining the fitting model and facilitating sub‐Ångstrom‐level depth resolution.^[^ ^60^ ^]^

A self‐consistent X‐ray magneto‐optical profile of the superlattice resulting from the fitting of three *q*_z_‐dependent XRMR spectra (non‐resonant, resonant, and XRMR‐XMCD) is depicted in Figure 1d. The profile represents the depth‐dependent variation (x‐axis) of the X‐ray optical constant Δβ_m_, which quantifies the magnitude of the modulation of the magnetic dichroism of the x‐ray absorption coefficient β at the resonant photon energy of the Mn *L*_3_ edge (642.5 eV). The all‐negative values are consistent with the traditional sign convention for representing XMCD signal at the *L*_3_ edge.

The immediate vicinity of the LaAlO_3_ substrate (at the distances from the substrate of approximately 0‐28.5 Å, as defined in Figure 1d) is occupied by the first 8 u.c.‐thick paramagnetic LaNiO_3_ layer. This layer exhibits no XMCD (Δβ_m_) signal, except for a narrow (≈2.39 Å) interdiffusion region where it interfaces with the first CaMnO_3_ layer. In the subsequent depth range (≈28.5–42.7 Å), we find the aforementioned 4 u.c.‐thick CaMnO_3_ layer, which exhibits a distinctive double‐peaked excursion in Δβ_m_. Such a magneto‐optical profile indicates a significant and highly localized increase in the net magnetic moment of Mn within the interfacial regions of the CaMnO_3_ layer. This LaNiO_3_/CaMnO_3_ bilayer profile is then replicated ten times, consistent with the nominal layering of the superlattice, with the exception of the uppermost CaMnO_3_ layer where only the bottom interface is ferromagnetic.

In order to better elucidate the local magnetic structure, in Figure 1e we plot a more detailed magneto‐optical profile of the CaMnO_3_ layer (as well as the two adjacent LaNiO_3_ layers) in the near‐central region of the superlattice. The figure illustrates that the maxima of the magnetic signal are located within the interfacial unit cells of CaMnO_3_ on both “ends” of the layer. The thickness of the ferromagnetic region in CaMnO_3_, as defined by the fitting model, is 3.40 ± 0.03 Å, which corresponds to approximately one pseudo‐cubic unit cell.^[^ ^44^, ^45^, ^61^ ^]^ However, there is a substantial broadening of the Δβ_m_ peak, characterized by the Névot‐Croce‐type interdiffusion,^[^ ^62^ ^]^ with a characteristic width of 2.39 ± 1.49 Å present on both sides of the ferromagnetic region. Notably, the tail of the Mn XMCD signal profile extends into the interfacial regions of the LaNiO_3_ films, a consequence of chemical interdiffusion (or roughness) between the CaMnO_3_ and LaNiO_3_ layers in the superlattice. This interfacial interdiffusion was detected and quantified using both resonant and non‐resonant soft X‐ray reflectivity measurements, as shown in Figure S2 of the Supporting Information. Consequently, although the ferromagnetism clearly originates in the interfacial unit cell of CaMnO_3_, the total extent of the ferromagnetic signal in a real‐life (non‐ideal) sample is on the order of 2‐3 u.c.

It is important to highlight that the minor asymmetries in the widths and amplitudes of the magnetic signal observed for the top and bottom interfaces in Figure 1d,e are typical for such superlattices. These differences have been previously ascribed to structural asymmetries, roughness, and interface reconstruction.^[^ ^60^, ^63^, ^64^ ^]^ It is also worth noting that a gradual degradation in the quality of the interfaces is observed in Figure 1d for several of the upper bilayers in the superlattice. It is characterized by the reduction of the maximum magnitudes of Δβ_m_ observed at the interfaces, as well as by the gradual merging of the two initially sharp interfacial peaks in Δβ_m_. This degradation is likely due to the cumulative increase in the number of defects, such as elemental interdiffusion and interface roughness/steps, which is typical for the PLD growth of superlattices consisting of many tens and/or hundreds of atomic layers.^[^ ^65^, ^66^ ^]^ Nevertheless, it is important to note that this effect becomes prominent only in the top 2‐3 bilayers and, even so, the observed ferromagnetic signal in the affected CaMnO_3_ layers still clearly originates from the interfacial regions.

As an additional reference measurement, we also performed XAS/XMCD characterization of the thinner (2LNO/4CMO) superlattice, in which the observed magnetic signal was considerably suppressed (see Figure S5 in the Supporting Information) compared to the 8LNO/4CMO sample. The residual magnetic signal, attributed to defect‐mediated phenomena, was too weak to allow for depth‐resolved (*q*_z_‐dependent) XRMR measurements.

In summary, we used a combination of XAS/XMCD spectroscopy and XRMR to measure the detailed magneto‐optical profile of the conducting 8LNO/4CMO superlattice and to show that the ferromagnetism in this system originates and resides mostly in the interfacial unit cells of CaMnO_3_. In the following section of this work, we demonstrate that this emergent interfacial phenomenon can be controlled using ultrafast high‐field (≈1 MV cm^−1^). THz E‐field pulses and discuss the interrelated electronic and magnetic processes driven by the excitation.

## 3 Ultrafast THz Dynamics and Control of the Interfacial Ferromagnetism

**Figure 2:**
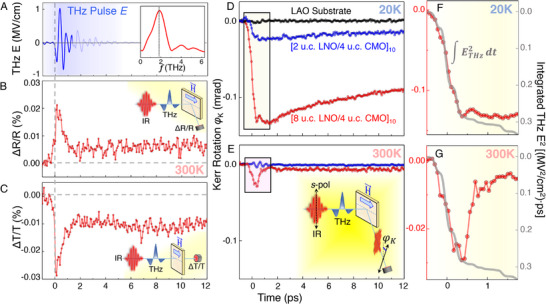
Electronic and magnetic dynamics in the LaNiO_3_/CaMnO_3_ superlattices induced by an ultrafast THz E‐field pulse. a) The THz pulse E‐field waveform, as measured and calibrated via standard electro‐optic sampling (EOS). Shaded (light blue) features after ≈1.5 ps are artefacts due to multiple reflections in the EOS crystal. The Fourier‐transformed pulse is shown in the inset. b,c) Ultrafast THz‐pump near‐IR‐probe (800 nm) time‐delay traces of the relative percent changes in b) reflectivity and c) transmissivit showing an ultrafast transient response followed by a long‐lived transient state characteristic of an ultrafast increase in the metallicity of the superlattice. Experimental geometries are shown in the insets. D,e) Time‐delay traces of the Kerr rotation ϕ_K_, as measured via tr‐MOKE for the two superlattice samples and the bare LaAlO_3_ substrate, d) below and e) above the Curie temperature (*T*_C_). Experimental geometry is shown in the inset (e). In addition to the expected temperature dependence of the magnetic response, the observed dynamics show strong LaNiO_3_ thickness dependence. The LaAlO_3_ substrate shows almost no measurable response to the THz pulse. f,g) The initial sub‐ps response of the delay traces follows in shape the integral of the square of the THz pulse waveform and is present in both f) below‐*T*_C_ and g) above‐*T*_C_ dynamics.

To induce and examine the ultrafast E‐field driven electronic and magnetic dynamics in the LaNiO_3_/CaMnO_3_ superlattices, we used single‐cycle THz pump pulses generated in an OH1 organic crystal through the optical rectification of 1300 nm radiation emitted by an optical parametric amplifier.^[^ ^67^ ^]^ **Figure** **2**a presents the resulting THz pulse, which exhibited a peak E‐field reaching 1 MV cm^−1^ and a central frequency of approximately 1.9 THz (see inset). The measurement and calibration of the pulse were conducted using standard electro‐optic sampling techniques.^[^ ^68^ ^]^

A portion of the 800 nm beam produced by the same Ti:sapphire amplifier served as a probe for investigating the electronic and magnetic dynamics. Three detection schemes, implemented in the same experimental setup and illustrated schematically in Figure 2a–e, were employed. The ultrafast reflectivity (at 45° incidence) and transmissivity (at normal incidence) measurements were utilized to examine the electronic response of the sample, while the time‐resolved magneto‐optical Kerr effect (tr‐MOKE) technique (at 45° incidence) was employed to probe the magnetic response at the interface. The measurements were conducted within an in‐plane applied magnetic field of approximately 0.47 T and in a closed cycle cryostat, enabling temperature control from 20 to 300 K.

SQUID magnetometry measurements on similar superlattices^[^ ^41^ ^]^ showed that at *T* = 10 K, the ferromagnetic signal saturates at in‐plane magnetic fields of approximately 0.15–0.4 T, depending on the thickness of the LaNiO_3_ layers. Therefore, we expect that in our experimental setup, with an applied magnetic field of 0.47 T and a base temperature of 20 K, the interfacial magnetic moments were fully saturated.

Figure 2b,c depicts time‐delay traces of the percent change in the near‐IR (*λ* = 800 nm) reflectivity (Δ*R*/*R*) and transmissivity (Δ*T*/*T*), respectively, recorded at room temperature (300 K) on the conducting 8LNO/4CMO superlattice. These delay traces represent the predominantly electronic (non‐magnetic) response of the system to the THz excitation, due to the above‐T_C_ temperature of the sample and the nominally magnetically insensitive detection modes (*R* and *T*).

The initiation of the electronic response is observed simultaneously with the onset of the THz excitation pulse shown in Figure 2a. As the data in Figure 2b,c demonstrate, at room temperature, the observed dynamics are dominated by processes occurring within the temporal window when the THz pulse is present in the sample (≈1 ps). Specifically, we observe a substantial increase in the relative optical reflectivity (Δ*R*/*R*) that is mirrored by a concomitant decrease in the relative transmissivity (Δ*T*/*T*). This suggests an electronic response consistent with non‐equilibrium Joule heating due to electron‐electron scattering^[^ ^48^ ^]^ as well as an E‐field‐driven modification of the electronic structure leading to a transient reduction of the near‐IR absorption, analogous to an ultrafast increase in the metallicity of the superlattice.^[^ ^20^, ^49^, ^50^ ^]^ These effects are observed simultaneously with the THz excitation pulse and diminish considerably once the THz pulse is no longer active within the sample (past *t* = 1 ps), giving way to a stable, long‐lived transient state with a partially collapsed bandgap (2 ps < *t* < 12 ps). This long‐lived partially metallic state, characterized by increased *R* and decreased *T*, persists for picoseconds after the THz pulse. This suggests a slow relaxation of the elevated lattice temperature associated with the partially metallic state.^[^ ^69^ ^]^ Similar dynamics have been observed recently in another strongly correlated oxide system, VO_2_, where THz pulses were shown to induce an insulator‐metal transition assisted by similar purely electronic mechanisms.^[^ ^20^ ^]^

It is important to note that, since CaMnO_3_ is an insulator with a measured bandgap of approximately 3.07 eV,^[^ ^70^ ^]^ the 800 nm (1.55 eV) probe is likely more sensitive to the partial collapse of the insulating bandgap in CaMnO_3_. As the LaNiO_3_ component of the superlattice is already metallic, its electronic state dynamics are likely more complex and would need to be studied using different techniques.

Figure 2d,e depicts the dynamic magnetic responses (Kerr Rotation ϕ_K_) of the 8LNO/4CMO and 2LNO/4CMO superlattices at two temperatures: 20 K (below *T*_C_) and 300 K (above *T*_C_), respectively. At first glance, it is immediately evident that the overall amplitudes of these responses are strongly temperature‐ and LaNiO_3_‐thickness dependent.

Specifically, the strongest magnetic response to the THz excitation is observed for the 8LNO/4CMO superlattice containing metallic LaNiO_3_ layers (red curve in Figure 2d), where interfacial ferromagnetic order is observed via XRMR‐XMCD at *T* = 20 K (see Figure 1). A much weaker (by ≈80%) magnetic response is observed for the 2LNO/4CMO superlattice containing insulating LaNiO_3_ layers (blue curve), where only a very weak residual ferromagnetic signal due to defect‐mediated phenomena is expected.

The ≈80% reduction in the signal is quantitatively consistent with the static SQUID magnetometry measurements on similar 8LNO/4CMO and 2LNO/4CMO samples by Flint et al.^[^ ^42^ ^]^ as well as XMCD measurements by Paudel et al.^[^ ^45^ ^]^ Such quantitative agreement with the prior studies of interfacial ferromagnetism in the LaNiO_3_/CaMnO_3_ superlattices is the first indication that the observed THz‐driven magnetic dynamics originate at the interface.

The second piece of evidence supporting the interfacial origin is observed in the temperature dependence of the overall amplitudes of the dynamic magnetic response, when comparing Figure 2d (below‐*T*_C_) and 2e (above‐*T*_C_). Namely, the tr‐MOKE effect is most pronounced and exhibits a rich multistep evolution for the 8LNO/4CMO superlattice in its ferromagnetic state at T = 20 K. Conversely, it is significantly weaker at *T* = 300 K, where it essentially mimics the purely‐electronic response, as seen in Δ*R*/*R* and Δ*T*/*T* in Figure 2b,c. These temperature‐dependent comparisons also confirm that potential non‐magnetic contributions, such as electro‐optic Kerr effects or transient birefringence, are negligible, since the signal above *T*_C_ is roughly 25 times weaker at delay times beyond 1 ps. Therefore, the observed tr‐MOKE response below *T*_C_ primarily reflects ultrafast magnetization dynamics driven by the THz excitation.

It is important to note that the initial sub‐ps response observed during the first 1 ps of the delay traces follows in shape the integral of the square of the THz pulse waveform and is present in both below‐*T*_C_ (Figure 2f) and above‐*T*_C_ (Figure 2g) dynamics. This finding confirms that the sub‐ps response includes a substantial electronic component and highlights that the observed polarization rotation arises from a combination of both electronic and magnetic contributions.

The underlying reason for the observed correlated behavior of the electronic and magnetic dynamics is the fact that the nonequilibrium current induced at the ferromagnetic interface by the THz pulse is, in fact, spin‐polarized^[^ ^71^ ^]^ and, therefore, becomes detectable not only in the *R* and *T*, but also in the Kerr rotation (ϕ_K_) channel.^[^ ^72^ ^]^ The attribution of sub‐picosecond demagnetization to THz‐driven spin‐polarized currents is consistent with a substantial body of prior experimental and theoretical work,^[^ ^37^, ^51^, ^52^, ^53^ ^]^ which collectively demonstrated that intense THz fields can drive ultrafast spin‐polarized currents in ferromagnetic systems, resulting in demagnetization on sub‐picosecond timescales. To further support this, our fluence‐dependent measurements shown in Figure S6 of the Supporting Information demonstrate that the amplitude of the response scales with the square of the THz peak E‐field, which is an expected functional dependence for energy dissipation due to scattering processes within a THz‐driven spin current.^[^ ^72^ ^]^

**Figure 3:**
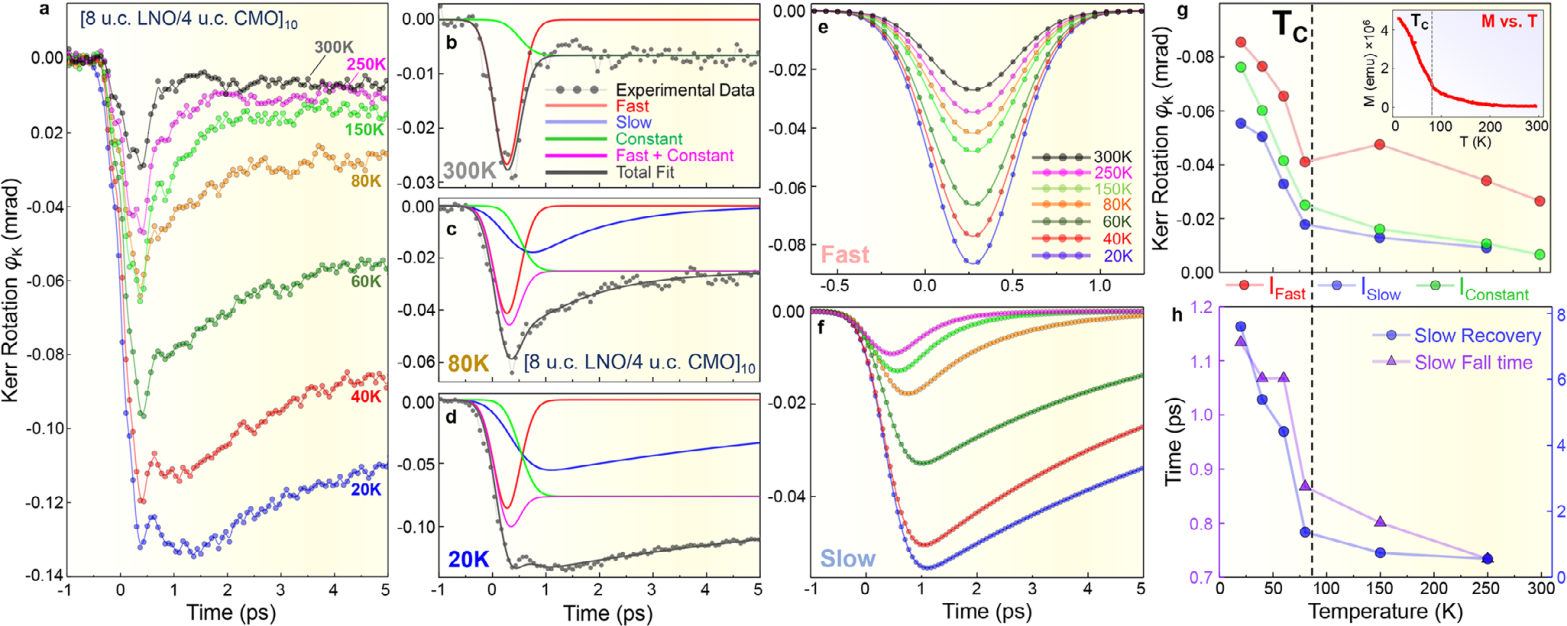
Temperature‐dependent tr‐MOKE and the origins of multiple dynamic magnetic responses. a) Temperature dependent tr‐MOKE delay traces recorded at seven temperatures varying from 300 to 20 K. b) Temporal decomposition of the room‐temperature (300 K) delay trace into the initial fast and quasiconstant components. c) Temporal decomposition of the delay trace recorded at *T*_C_ = 80 K requires an additional dynamic component characterized by a slower fall time and consequent exponential recovery (blue curve). d) Temporal decomposition of the 20 K delay trace requires all three dynamic components. e) All the temperature‐dependent fits of the fast dynamic component, exhibiting uniform temporal behavior. f) Temperature‐dependent fits of the slow dynamic component, which exhibits onset at 250 K and grows at the onset of interfacial ferromagnetism (*T* < 80 K). g) Amplitudes and the h) characteristic time constants of the dynamic components of the tr‐MOKE delay traces plotted as functions of temperature. All parameters exhibit a sharp decline and a change in slope at 80 K, marking the critical temperature (*T*_C_) for the interfacial magnetic order, consistent with static SQUID magnetometry measurements shown in the inset (Reproduced with permission.^[^ ^74^ ^]^ Copyright 2013, American Physical Society).

**Figure 4:**
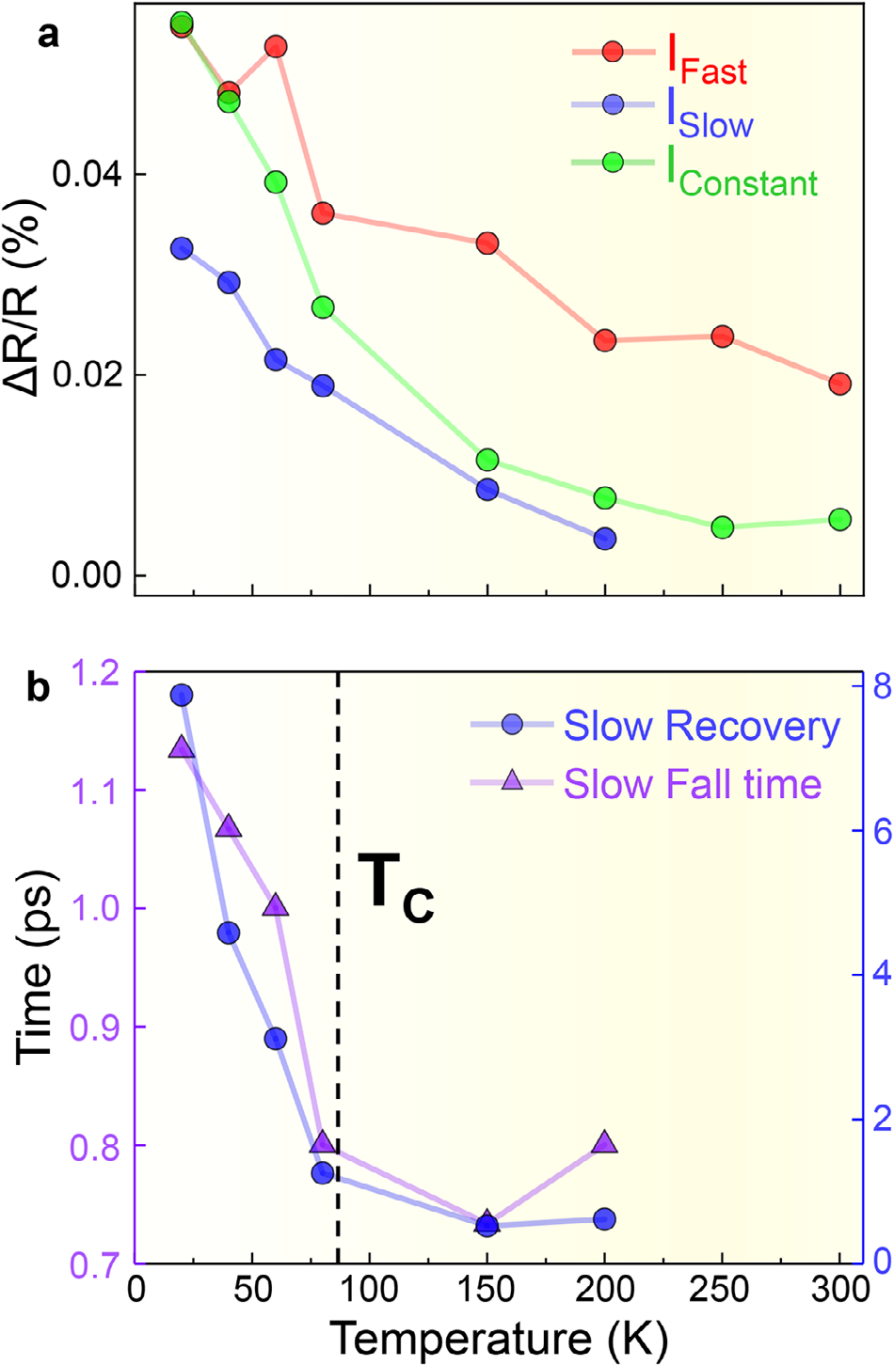
Temperature dependent ultrafast reflectivity and the electronic response of the superlattice. a) Temporally decomposed amplitudes of the fast, slow, and quasiconstant dynamic components observed in the ultrafast Δ*R*/*R* response plotted as function of temperature. A monotonic decline in the intensities of all the components is observed with rising temperature. b) The temperature dependence of the ultrafast Δ*R*/*R* time constants of the slow feature exhibits signatures of the magnetic response of the system due to the strong correlation between the charge and spin dynamics in this system.

The final key observation from Figure 2d is that, at the temperatures below *T*_C_, an additional dynamic component characterized by a slower fall time (≈1.1 ps) and consequent exponential recovery arises in the tr‐MOKE signal. This component is most evident in the red time‐trace (8LNO/4CMO) in Figure 2d. In order to investigate its temperature dependence and ascertain its origin, we carried out systematic temperature‐dependent tr‐MOKE and near‐IR transient reflectivity measurements, which are shown and analyzed in **Figures** **3** and **4** below.

Figure 3a shows the time‐resolved magneto‐optical response of the sample measured via tr‐MOKE at seven temperatures varying from 300 K (room temperature) to 20 K. Each data point represents an average of 150 laser shots, repeated over 4 independent time‐delay scans, with several key measurements reproduced over a 42 d period to ensure stability and reproducibility. The initial sub‐ps response to the THz excitation observed during the first 1 ps of the delay traces is observed for all temperatures. For brevity, from now on, we will refer to this dynamic component as the “fast” peak. In addition to this feature, the 300 K delay trace (black curve), which essentially mimics the purely‐electronic response as observed in Δ*R*/*R* and Δ*T*/*T*, also exhibits the long‐lived transient state characterized by the stable plateau in the Kerr rotation (ϕ_K_) from ≈1 to 5 ps.

Due to its simplicity, the 300 K delay trace can be effectively fitted using two functional components—a Gaussian lineshape representing the fast peak (red curve in Figure 3b) and a quasiconstant component characterized by a step function broadened by the width of the THz pulse, ≈1 ps (shown as the green curve). The total fit to the data is shown as a solid dark gray curve in Figure 3b. Although this fitting model does not consider the fine (sub‐ps) structure of the THz pulse (see Figure 2a), it effectively accounts for the major features of the delay trace corresponding to different THz‐induced physical phenomena in the sample. This model was recently used to describe the ultrafast photoinduced insulator‐metal transition in SmNiO_3_.^[^ ^73^ ^]^

It should be noted that the tr‐MOKE signal observed above the magnetic transition temperature (*T*_C_ = 80 K) is predominantly electronic in origin and may arise from a combination of effects such as electronic thermal diffusion, thermally induced strain, charge redistribution, or electro‐optic Kerr contributions.

As temperatures decrease toward *T*_C_ = 80 K, an additional dynamic component characterized by a slower fall time and consequent exponential recovery emerges, becoming pronounced precisely at *T*_C_. Incorporating this component into our fitting model is essential for accurately describing the shape of the 80 K delay trace. It is shown as the blue curve in Figure 3c. This adjustment is also necessary for fitting all lower‐temperature delay traces, as illustrated by the 20 K data in Figure 3d. Henceforth, we will refer to it as the “slow” component that arises and grows at the onset of interfacial ferromagnetism in the LaNiO_3_/CaMnO_3_ superlattice.

Figure 3e,f depicts all the temperature‐dependent fits of the fast and slow components, respectively. For the fast peak, the Gaussian width and the center position (270 fs) characterizing the fall time of the effect are independent of temperature. This is expected because of the electronic nature of the underlying physical effect, which involves non‐equilibrium Joule heating and THz‐field‐induced spin‐polarized currents leading to the ultrafast demagnetization of the ferromagnetic interfacial layer. Notably, conventional spin‐conserving scattering cannot account for the ultrafast demagnetization, as it lacks a pathway for angular momentum transfer to the lattice. In contrast, Elliott–Yafet‐type spin‐flip scattering of THz‐induced, non‐equilibrium spin‐polarized carriers efficiently transfers angular momentum out of the spin subsystem to the lattice, thereby reducing the net magnetization.^[^ ^37^ ^]^ Consequently, the temporal profile of this dynamic component is exclusively determined by the shape (width and time‐zero) of the THz pulse.

On the other hand, the amplitude of the fast peak displays a pronounced temperature dependence, as depicted in Figure 3g (red symbols). It is readily apparent that this behavior aligns with the static SQUID magnetometry (*M* vs *T*) measurements, revealing a sharp decline and a change in slope at 80 K, marking the critical temperature (*T*_C_) for the interfacial magnetic order (see inset in Figure 3g as well as Figure S4 in the Supporting Information). The time‐resolved data also accurately captures the presence of some residual magnetic signal observed between *T*_C_ and 200–250 K, which has been documented in prior studies and attributed to the presence of multiple competing magnetic interactions in the superlattice.^[^ ^74^ ^]^ Thus, the agreement between static magnetometry and dynamic tr‐MOKE data provides additional compelling evidence that the THz pulse directly influences the interfacial ferromagnetic order on the ultrafast scale.

The amplitudes of the *slow* and the quasiconstant components similarly exhibit temperature dependence that is consistent with the magnetometry (*M* vs *T*) measurements, showing an abrupt change in slope at *T*_C_ = 80 K and some residual signal up to 200–250 K (blue and green symbols in Figure 3g). Importantly, the *slow* component also exhibits qualitative temperature‐dependent changes in its fall time (purple symbols in Figure 3h) as well as its exponential recovery constant (blue symbols). This stands in stark contrast to the temperature‐independent temporal response of the fast time‐zero component, as evident when comparing individual time‐delay traces in Figure 3e,f.

The absence of the slow dynamic component in the tr‐MOKE signal at room temperature (see Figure 3b), followed by its emergence between 200 and 250 K and subsequent rapid increase below *T*_C_ = 80 K (see Figure 3g), confirms its magnetic origin. Simultaneously, qualitative temperature‐dependent changes in the time constants (fall time and exponential recovery) suggest the transfer of spin angular momentum to the lattice (and, subsequently, back to the spin subsystem). This is in agreement with several recent works that have reported intriguing observations of the transfer of angular momentum between spin and lattice systems, and vice versa.^[^ ^75^, ^76^, ^77^, ^78^, ^79^, ^80^, ^81^ ^]^ Our results are consistent with these magnetoelastic mechanisms and align with the growing body of evidence that angular momentum transfer can indeed flow from the spin subsystem to the lattice and subsequently back to the electronic magnetization. Specifically, the transient increase (recovery) in magnetization can be explained by the transfer of angular momentum from the lattice back into the electronic magnetization, a process that has been observed in comparable oxide‐based multilayers.^[^ ^80^ ^]^ It should be noted that, in addition to magnetoelastic coupling, alternative mechanisms such as ultrafast magnon generation and subsequent angular momentum transport^[^ ^82^, ^83^ ^]^ may also contribute to the observed long‐timescale recovery dynamics.

However, at higher temperatures, the increased phonon population enhances the coupling between spin and lattice degrees of freedom, making the magnetoelastic interaction more efficient and, thus, faster. Conversely, at lower temperatures, reduced phonon availability limits interactions between spin and lattice, slowing down magnetoelastic coupling and affecting both the onset and recovery rates. Thus, the observed temperature‐dependent variations in time constants (see Figure 3h) can be explained by considering the influence of phonon density on the efficiency of spin‐phonon interactions.

We note that this bidirectional angular momentum transfer between the spin and lattice subsystems can be naturally understood within the framework of conventional microscopic models, such as the Elliott–Yafet spin‐flip scattering mechanism and the microscopic three‐temperature model (M3TM). In this picture, the THz excitation drives ultrafast demagnetization via spin‐lattice angular momentum transfer mediated by electron–phonon spin‐flip processes, after which the lattice, acting as an intermediate angular momentum reservoir, returns part of this angular momentum to the spin system on tens‐of‐picosecond timescales. This mechanism is expected to remain operative in quasi‐2D systems (see, e.g., ref.[84]), since Elliott‐Yafet spin‐lattice scattering involves localized angular momentum transfer that is largely unaffected by dimensional confinement. Overall, this interpretation positions our findings within the broader theoretical and experimental context of angular momentum exchange among spin, electron, and lattice subsystems, as established in prior studies of both metallic and oxide‐based systems.^[^ ^75^, ^76^, ^77^, ^78^, ^79^, ^80^, ^81^ ^]^

## 4 Discussion

In summary, our time‐ and temperature‐dependent THz‐pump tr‐MOKE measurements helped identify and disentangle several different ultrafast responses of the quasi‐2D interfacial ferromagnetic state at the LaNiO_3_/CaMnO_3_ interface to an intense single‐cycle THz electric‐field pulse—a sub‐ps time‐zero response consistent with ultrafast demagnetization driven by the nonequilibrium THz‐induced spin‐polarized currents, and multi‐ps‐scale change in the magnetic state of the superlattice due to the transfer of spin angular momentum to the lattice. These magnetic and magnetoelastic responses are observed concomitantly with the purely‐electronic effects, such as the non‐equilibrium Joule heating and an ultrafast increase in the metallicity of the superlattice. These electronic and magnetic phenomena appear to be strongly interconnected, which suggests an effective approach for controlling interfacial magnetism via ultrafast THz E‐field pulses.

In order to gain better insight into the electronic dynamics initiated by the THz pulse, we have carried out ultrafast temperature‐dependent THz‐pump‐near‐IR (800 nm) reflectivity measurements of the 8LNO/4CMO superlattice in the same experimental setup. These measurements are complementary to the tr‐MOKE experiments, as transient reflectivity (Δ*R*/*R*) and tr‐MOKE signals are well established as probes of the diagonal and off‐diagonal components of the dielectric tensor, respectively. While Δ*R*/*R* traces the evolution of the time‐reversal‐invariant diagonal components, reflecting purely electronic and structural effects, the MOKE response arises from changes in the off‐diagonal components that break time‐reversal symmetry and therefore signify a magnetic origin.^[^ ^85^ ^]^

Due to the strong correlation between the THz‐induced magnetic and electronic dynamics in this system, similar features (fast and slow) can also be observed in the electronic response of the superlattice measured via transient near‐IR reflectivity (see Figure S7 in the Supporting Information). These components were quantitatively analyzed using the same fitting scheme as that employed for the tr‐MOKE spectra, consisting of fast, slow, and quasiconstant contributions. However, the temperature dependence of the amplitudes of the fast, slow, and quasiconstant dynamic Δ*R*/*R* responses does not align with the static SQUID magnetometry (*M* vs *T*) measurements. Specifically, the Δ*R*/*R* versus *T* curves (see Figure 4a) exhibit no change in slope at the *T*_C_ (80 K). Instead, all three features exhibit a monotonic decline in intensity with rising temperature, as expected due to the temperature‐dependent decline in conductivity (see electronic transport measurements in Figure S3 of the Supporting Information). Thus, these additional measurements suggest that the combination of ultrafast Δ*R*/*R* and tr‐MOKE measurements can help decouple the electronic and magnetic responses of an interfacial ferromagnetic state.

It is important to point out that the signatures of the magnetic response of the system persist in the temperature dependence of the Δ*R*/*R* time constants (fall time and the exponential recovery constant) of the *slow* feature due to its magnetic origin (see Figure 4b). Such a correlation between the electronic and magnetic responses of the system offers a way of probing magnetic dynamics of the interfacial ferromagnetic state via ultrafast THz‐pump near‐IR reflectivity probe techniques.

The future undoubtedly holds many more exciting developments in the field of ultrafast THz spintronics, with the imminent advances in the ultrafast element‐ and spin‐resolved probes of THz‐driven dynamics, such as tr‐ARPES^[^ ^86^ ^]^ and x‐ray free‐electron laser‐based techniques.^[^ ^87^, ^88^ ^]^ These probes of ultrafast electronic and magnetic dynamics will be vital in revealing the full time‐, spin‐, and momentum‐resolved picture of the fundamental interaction at the interfaces of strongly correlated and quantum materials.

## 5 Experimental Section

### XAS and XMCD

The XAS and XMCD measurements were conducted using the Vector Magnet endstation at the high‐resolution (100 meV) Magnetic Spectroscopy and Scattering beamline 4.0.2 at the Advanced Light Source.^[^ ^57^ ^]^ The measurements were performed in the bulk‐sensitive luminescence yield (LY) detection mode at a temperature of *T* = 20 K and with an in‐plane magnetic field of 0.5 T. The X‐ray beam (100 µm diameter) was incident on the sample at an angle of 30 degrees, as measured from the sample plane. Multiple measurements were carried out at various locations on the sample to rule out the possibility of X‐ray beam damage.

### X‐Ray Magnetic Reflectivity (XRMR)

The magneto‐optical profiling of the superlattice via XRMR was carried out using the resonant X‐ray scattering end station at the same beamline. The samples were aligned with their surface‐normal in the scattering plane and measured at *T* = 20 K and in an in‐plane magnetic field of 0.1 T. The measurements were carried out in the specular *θ*–2*θ* reflection geometry at both resonant (Mn *L*_3_ edge) and non‐resonant (620 eV) photon energies using circularly polarized X‐rays. The data were fitted using the X‐ray reflectivity analysis program ReMagX^[^ ^59^ ^]^ that uses an algorithm based on the Parratt formalism^[^ ^89^ ^]^ and the Névot–Croce interdiffusion approximation.^[^ ^62^ ^]^ For the non‐resonant spectrum fitting, only the thicknesses of the CaMnO_3_ and LaNiO_3_ layers and the interdiffusion lengths between them were allowed to vary. For the resonant spectrum fitting, the variables included the thickness and roughness of the interfacial magnetic layer and the X‐ray optical constant Δβm, which quantifies the magnitude of the modulation of the magnetic dichroism of the X‐ray absorption coefficient β. The resonant X‐ray optical constants required for the calculations were obtained by performing a Kramer‐Kronig analysis of the XAS data and later optimized during the fitting of the resonant reflectivity spectra.

### Ultrafast THz Measurements

The ultrafast THz‐pump near‐IR probe measurements were conducted at Stockholm University's THz laboratory. The experiment utilized intense single‐cycle THz electric fields, generated through optical rectification of 1300 nm pulses in an OH1 organic crystal. The generated THz field was modulated using a mechanical chopper at 500 Hz (half the laser repetition rate, 1 kHz), allowing for pump‐on and pump‐off measurements. In all experiments detailed in the main text, *s*‐polarized THz pump pulses and *p*‐polarized 800 nm probe pulses with a nominal pulse duration of 40 fs were employed. The measurements used a balanced detection scheme for tr‐MOKE measurements, where photodiodes were balanced using a half‐wave plate. For ultrafast reflectivity and transmittivity measurements, we used an unbalanced differential detection without any waveplates. All measurements were carried out within a cryostat, affording the flexibility to adjust temperatures within the range of 4 to 300 K, enabling temperature‐dependent investigations. A custom‐made sample holder, equipped with permanent magnets of 0.47 T field strength, was used inside the cryostat to secure the sample. In the fluence‐dependent measurements mentioned in Figure S7 (Supporting Information), two wire‐grid polarizers were employed to control the THz pump fluence. The THz pump field was characterized through free space electro‐optic sampling in a 50 µm thick GaP crystal.

### HAXPES

The bulk‐sensitive core‐level and valence‐band HAXPES measurements were conducted at the P22 beamline^[^ ^90^ ^]^ of the PETRA III synchrotron at DESY. The photon energy was set at 6.0 keV. At this energy, the values of the inelastic mean‐free paths of the photoelectrons in CaMnO_3_ and LaNiO_3_ are estimated to be approximately 87 Å and 71 Å, respectively, with the maximum probing depth being roughly three times these values.^[^ ^91^ ^]^ The total experimental energy resolution (380 meV at the analyzer pass energy of 50 eV) and the position of the zero binding energy were determined by measuring the Fermi edge of a standard Au sample. The measurements were carried out at the sample temperature of approximately 77 K. Additional supporting references are listed in the Supporting Information.^[^ ^92^, ^93^, ^94^, ^95^, ^96^, ^97^, ^98^ ^]^

## Conflict of Interest

The authors declare no conflict of interests.

## Supporting information



Supporting Information

## Data Availability

The data that support the findings of this study are available from the corresponding authors upon reasonable request.
