# Supplementary material for: Ultrafast Terahertz Field Control of the Emergent Magnetic and Electronic Interactions at Oxide Interfaces
Source: Adv Mater. 2025 Nov 24;38(8):e12328. doi: 10.1002/adma.202512328 (PMC12878812; doi:10.1002/adma.202512328)
Supplement: Supplementary file 1 — Supporting Information [file ADMA-38-e12328-s001.pdf]

ADVANCED MATERIALS

Supporting Information

for *Adv. Mater.*, DOI 10.1002/adma.202512328

Ultrafast Terahertz Field Control of the Emergent Magnetic and Electronic Interactions at Oxide Interfaces

Abigail M. Derrico, Martina Basini, Vivek Unikandanunni, Jay R. Paudel, Mikhail Kareev, Michael Terilli, Tsung-Chi Wu, Afnan Alostaz, Christoph Klewe, Padraic Shafer, Andrei Gloskovskii, Christoph Schlueter, Claus M. Schneider, Jak Chakhalian, Stefano Bonetti and Alexander X. Gray**

Supporting Information

Ultrafast Terahertz Field Control of the Emergent Magnetic and Electronic Interactions at Oxide Interfaces

Abigail M. Derrico,[1,2] Martina Basini,[3,4] Vivek Unikandanunni,[3,5] Jay R. Paudel,[1] Mikhail Kareev,[6] Michael Terilli,[6] Tsung-Chi Wu,[6] Afnan Alostaz,[7] Christoph Klewe,[8] Padraic Shafer,[8] Andrei Gloskovskii,[9] Christoph Schlueter,[9] Claus M. Schneider,[7] Jak Chakhalian,[6] Stefano Bonetti,[3,10,]* and Alexander X. Gray[1,]*

[1] Department of Physics, Temple University, Philadelphia, Pennsylvania 19122, USA

[2] Department of Physics, University of California, Berkeley, California 94720, USA

[3] Department of Physics, Stockholm University, 10691 Stockholm, Sweden

[4] Physics Department, ETH Zurich, 8093 Zürich, Switzerland

[5] Institute of Applied Physics, University of Bern, 3012 Bern, Switzerland

[6] Department of Physics and Astronomy, Rutgers University, Piscataway, New Jersey 08854, USA

[7] Peter Grünberg Institut (PGI-6), Forschungszentrum Jülich GmbH, D-52425 Jülich, Germany

[8] Advanced Light Source, Lawrence Berkeley National Laboratory, Berkeley, California 94720, USA

[9] Deutsches Elektronen-Synchrotron, DESY, 22607 Hamburg, Germany

[10] Department of Molecular Sciences and Nanosystems, Ca' Foscari University of Venice, 30172 Venice, Italy

* stefano.bonetti@unive.it, axgray@temple.edu

1. Structural Characterization via X-ray Diffraction (XRD) and Soft X-ray Reflectivity (SXR)

Supporting Figures S1a and S1b illustrate the high-resolution θ-2θ diffraction spectra of the 2LNO/4CMO and 8LNO/4CMO superlattices, respectively. The $LaAlO_3$ substrate peak appears at 2θ = 48° for both samples, in line with previous studies [42,44,45]. The 0th-order superlattice peak (SL_0) for the thinner sample (2LNO/4CMO) is obscured by the substrate peak (Fig. S1a), aligning with earlier investigations of analogous LNO/CMO superlattices [42,45]. In the case of the thicker superlattice (8LNO/4CMO) (Fig. S1b), the same peak is observed at a slightly lower angle (47°), consistent with the aforementioned studies [42,45]. The two 1st-order superlattice peaks (SL_{-1} and SL_{+1}) for the thinner sample (2LNO/4CMO) are observed at symmetric angular positions of 44° and 52°, approximately ±4° from the 0th-order (SL_0) peak. Conversely, for the 8LNO/4CMO superlattice, the two 1st-order superlattice peaks

(SL_{-1} and SL_{+1}) are closer to the 0th-order (SL_0) peak (±2.15°), as expected from basic considerations [42,61,74].

All superlattice peaks exhibit shapes characteristic of high-quality single-crystalline superlattices. Additionally, the spectrum for the thinner superlattice (2LNO/4CMO) displays pronounced SL thickness fringes. The number of aforementioned fringes (8) corresponds to the expected number (P − 2 = 8) of superlattice periods (P = 10). In the case of the thicker superlattice (8LNO/4CMO), the SL thickness fringes appear much closer and merge together, in line with prior studies [45,74]. A more comprehensive structural characterization of the thicker superlattice (8LNO/4CMO), central to this study, was conducted using synchrotron-based resonant and non-resonant soft X-ray reflectivity (SXR), and the details are described below.

Supporting Figures S2a and S2b display q_z-dependent specular soft X-ray reflectivity (SXR) spectra recorded using photon energies corresponding to off-resonant (620 eV) and resonant (Mn L_3 at 642.7 eV) conditions, respectively. The spectra cover a broad range of q_z (0.05 - 0.35 1/Å), encompassing both the 1st-order and 2nd-order Bragg conditions (at ~0.15 and ~0.30 1/Å, respectively), and thus provide detailed depth-resolved information on both the layering and interfacial structure of the sample [47,59,60]. The experimental data (red curves) were self-consistently fitted with the SXR analysis program ReMagX [59], employing an algorithm based on the Parratt formalism [89] and the Névot–Croce interdiffusion approximation [62]. In the model, only the thicknesses of the $CaMnO_3$ and $LaNiO_3$ layers, and the interdiffusion lengths between them (interface roughness), were allowed to vary. The resonant X-ray optical constants required for fitting the on-resonance data were obtained through a Kramer-Kronig analysis of the XAS data in Figure 1a of the main text.

The blue-colored spectra depicted in Supporting Figures S2a and S2b illustrate optimal theoretical fits to the experimental data, while Figures S2c and S2d show the resulting X-ray optical profile of the 8LNO/4CMO superlattice. The aforementioned superlattice profile is represented as the depth-dependent variation of the X-ray absorption coefficient β at the photon energy corresponding to the Mn L_3 absorption edge. The maxima in such an element-selective (Mn) absorption profile correspond to the depth-resolved positions of the $CaMnO_3$ layers, and the minima to the positions of the $LaNiO_3$ layers, where the Mn element is absent. The individual layer thicknesses obtained from the X-ray optical fitting are 28.46 ± 0.22 Å for $LaNiO_3$ and 14.25 ± 0.08 Å for $CaMnO_3$. These values correspond to approximately 7.5 u.c. of $LaNiO_3$ and approximately 4 u.c. of $CaMnO_3$, using lattice constants from prior studies [44,45,61,92,93]. The average interface roughness (interdiffusion) is 3.93 ± 0.54 Å, which equates to approximately one

unit cell of a typical perovskite oxide and aligns with typical high-quality layer-by-layer growth [55]. This observed interface roughness may account for the slightly underestimated (by 0.5 u.c.) average thickness of the $LaNiO_3$ layer (nominally 8 u.c.).

2. Chemical, Electronic, and Magnetic Characterization

The nominal chemical composition of the superlattices was confirmed through bulk-sensitive HAXPES measurements conducted at the P22 beamline [90] of the PETRA III synchrotron at DESY. Supporting Figure S3a presents wide-energy range HAXPES survey spectra for the 8LNO/4CMO (red line) and 2LNO/4CMO (blue line) superlattices. The presence of all expected elements (Ca, Mn, O, La, Ni, and C from the surface-adsorbed contaminant C/O layer) is verified by corresponding core-level peaks. The greater total thickness of the $LaNiO_3$ layers in the red 8LNO/4CMO spectrum is evidenced by the higher relative intensities of the La 3*d* and 4*p* peaks, compared to the blue 2LNO/4CMO spectrum.

To investigate the thickness-dependent variation in the valence-band electronic structure of $LaNiO_3$ and its impact on the resistivity of the superlattices, we employed a combination of bulk-sensitive valence-band HAXPES spectroscopy and electronic transport measurements. Supporting Figure S3b displays the experimental valence-band spectra of the 8LNO/4CMO superlattice (red line) and the 2LNO/4CMO superlattice (blue line). The corresponding temperature-dependent sheet resistance curves, measured using the standard van der Pauw method, are shown in the inset.

The near-Fermi-level region of the red 8LNO/4CMO superlattice spectrum exhibits two prominent features at 0.3 eV and 1.0 eV which, based on prior studies, correspond to the strongly-hybridized Ni 3*d* e_g and t_{2g} states, respectively [45,94,95]. In line with earlier research on the thickness-dependent metal-insulator transition in $LaNiO_3$ [45,94,95], the superlattice containing below-critical-thickness $LaNiO_3$ layers (2LNO/4CMO) shows a considerable suppression of these near-Fermi-level electronic states, resulting in an approximately two orders of magnitude enhancement in sheet resistivity.

In the context of interfacial ferromagnetism, in the thicker 8LNO/4CMO superlattice, the aforementioned Ni 3*d* states facilitate charge transfer from Ni to the interfacial Mn sites, creating an electronic environment that stabilizes the ferromagnetic state mediated by the double exchange interaction [41,42,44,45,61,74]. The depletion of the Ni 3*d* e_g states in the thinner 2LNO/4CMO superlattice leads to the metal-insulator transition in $LaNiO_3$ and the concomitant suppression of the interfacial ferromagnetic state in $CaMnO_3$.

Below, we probe the emergent interfacial ferromagnetic state in the 8LNO/4CMO superlattice using temperature-dependent SQUID magnetometry and compare our results with a prior study of a similar superlattice [74].

Supporting Figure S4 shows the temperature dependence of the Mn magnetic moment from 30 to 300 K after field-cooling in 7 T with a 2 T warming field (red symbols, left *y*-scale). The magnetic moment was normalized to the estimated number of interfacial Mn ions in the superlattice. The observed saturated magnetic moment of approximately 1.1 μ_B per interfacial Mn at T = 20 K is in excellent agreement with the previously reported value of ~1 μ_B per interfacial Mn [42]. A standard background subtraction procedure described in Ref. [96] was utilized to isolate the superlattice contribution to the magnetic signal and to account for the substrate ($LaAlO_3$) contribution.

We compare our data to the M vs. T curve obtained in a prior study [74] on a similar (6LNO/4CMO) superlattice (blue curve, right *y*-axis). This data exhibits a slightly better signal-to-noise ratio due to the lower value (0.5 T) of the warming field used during the measurement.
This allows for the observation of the finer details of the temperature dependence of the magnetic moment, such as the slow onset of the ferromagnetic signal between 250 and 200 K, as well as the abrupt change of the slope at T_C = 80 K. In summary, the two datasets exhibit good qualitative agreement. This agreement becomes quantitative when we also consider the observed values of the saturated magnetic moment at high fields (1.1 μ_B/Mn vs. 1 μ_B/Mn) [42].

As an additional reference measurement, we performed XAS/XMCD characterization on the thinner 2LNO/4CMO superlattice, where the observed magnetic signal was considerably suppressed compared to the 8LNO/4CMO sample (see Fig. S5 below). The bulk-sensitive Mn $L_{2,3}$ XAS spectrum (top panel) was measured in luminescence detection mode at T = 20 K, probing the entire sample depth. The corresponding XMCD spectrum (bottom panel), recorded in an in-plane magnetic field of 0.5 T, revealed only a weak residual magnetic signal, attributed to defect-mediated phenomena. This signal was insufficient to enable depth-resolved (q_z-dependent) XRMR measurements.

3. The X-ray Optical Modeling of the SXR and XRMR-XMCD Data

For the X-ray optical fitting of the SXR data (Figure S2) and XRMR data (Figure 1), we employed a three-step hierarchical fitting process within the framework of the ReMagX analysis software [59]. The same process was employed in a recent study of $CaMnO_3$/$CaRuO_3$ magnetic interfaces [97]:

Step 1: Non-Resonant SXR Fit

The *non-resonant* SXR data were first used to determine the layer thicknesses and roughnesses. The non-resonant optical constants (δ and β) were fixed to tabulated values from the LBNL CXRO database, which are accurate for non-resonant energies and widely used for SXR fitting. To ensure structural uniformity, the CMO and LNO layer thicknesses were initially kept identical across the superlattice to obtain an approximate fit and a set of starting parameters. They were then allowed to vary only systematically by 0.25% relative to the preceding layer. This slight, systematic variation in thickness is typical for PLD-grown superlattices with many bilayer repetitions and has been investigated in depth in prior studies (e.g., Ref. [98]). This step established the structural model used in all subsequent fits.

Step 2: Resonant SXR Fit

The thicknesses and roughnesses determined from the non-resonant fit were fixed. Only the resonant δ and β values were fine-tuned, starting from Kramers-Kronig-derived estimates obtained from XAS, to match the experimental resonant SXR data. This optimization is always necessary for several reasons: (i) XAS and SXR are distinct techniques employing different detection schemes and instruments, so some differences in the derived x-ray optical properties are expected; and (ii) δ and β values from Kramers-Kronig analysis depend on data post-processing (e.g., normalization, background subtraction) and on the energy range used for the Hilbert transformation. Therefore, a perfect correspondence between XAS- and SXR-derived constants is not expected, and some minor, tightly constrained optimization is required. This step ensured that the resonant optical constants (δ and β) for CMO are accurate and further confirmed the accuracy of the structural model established in Step 1.

Step 3: XRMR–XMCD Fit

The final magnetic fit was the most constrained. All structural (thickness, roughness) and non-magnetic optical parameters were fixed from Steps 1 and 2. The magnetic layer thickness was varied only in one representative layer, and this value was then propagated and constrained to be identical across all equivalent magnetic layers in the superlattice. The magneto-optical constants (δ_m and β_m) of the first magnetic layer were similarly varied and subsequently constrained to be the same for all magnetic layers. The extent of magnetic interdiffusion within the artificially sliced CMO layer was also varied. The apparent variations in the amplitudes of the magneto-optical constants (δ_m and β_m) arise from the Nevot–Croce-type broadening of the individual magnetic layers.

Overall, this hierarchical fitting strategy minimized the number of free parameters and ensured that all refinements remained physically meaningful.

4. Fluence-Dependent THz-pump tr-MOKE Measurements (8LNO/4CMO sample, T = 20 K)

Supporting Figure S6 below presents a series of fluence-dependent THz-pump tr-MOKE time-delay traces recorded on the 8LNO/2CMO superlattice at T = 20 K. The THz-pump excitation strengths are expressed in terms of the peak E-field of the THz pulse, measured and calibrated using standard electro-optic sampling. The fluence (peak E-field) was varied from the maximum value of 1.03 MV/cm to the minimum value of 0.12 MV/cm using a pair of rotatable THz wire-grid polarizers. The individual time-delay traces recorded at several values of the THz peak E-field within this range are displayed in panel a). Panel b) presents the average amplitudes of the magnetic response (Kerr rotation angle ϕ_K) for the 'fast' time-zero response (peak), obtained by averaging over several time delays around the 'fall time' of approximately +250 fs. The solid black curve represents the best fit of the experimental data to a parabola.

5. Ultrafast Temperature-Dependent THz-pump near-IR Reflectivity (ΔR/R) Measurements

Supporting Figure S7 displays the results of additional ultrafast temperature-dependent THz-pump near-IR (800 nm) reflectivity probe measurements of the 8LNO/4CMO superlattice in the same experimental setup. Panel a) shows the time-resolved electronic response of the sample measured via relative (%) change in transient IR reflectivity (ΔR/R) at several temperatures varying from 300 K (room temperature) to 20 K. Panels b)-d) illustrate temporal decomposition of the delay traces recorded at room temperature (b), T_C = 80 K (c), and T = 20 K (d) into three distinct dynamic components (fast, quasiconstant, and slow) using the fitting procedure described in the main text. Panels e) and f) show all the resultant temperature-dependent fits of the 'fast' and 'slow' dynamic temporal components, respectively. Results of the analyses of the amplitudes and time constants are shown (and discussed) in Figure 4 of the main text.

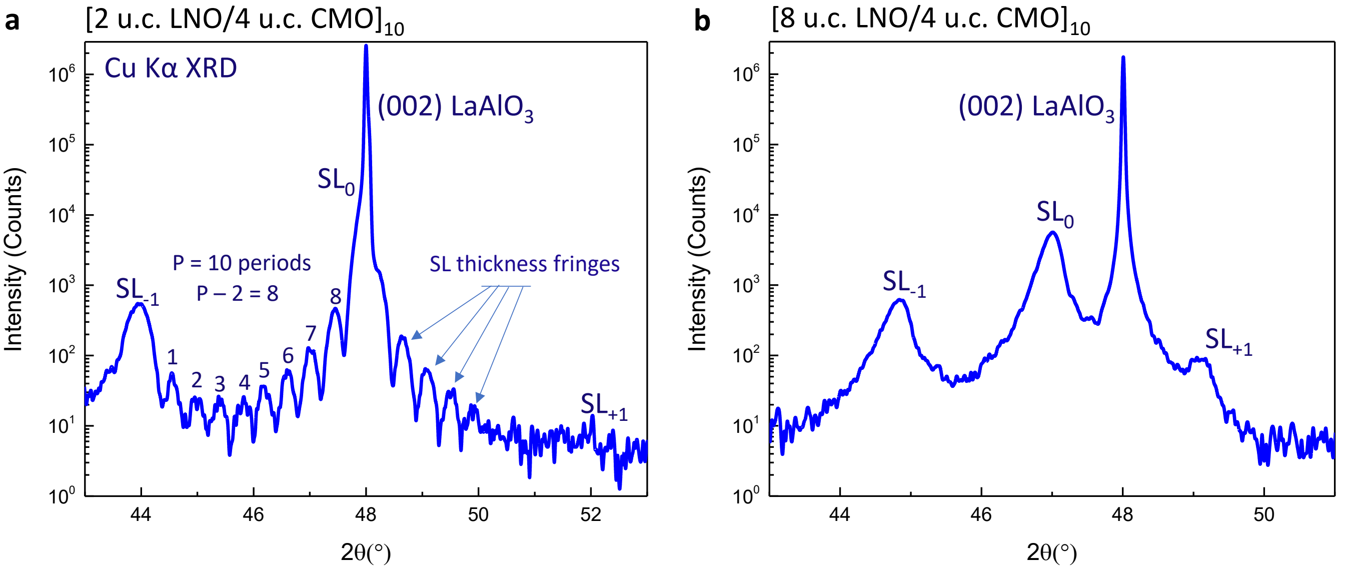

Figure S1. Structural characterization via X-ray diffraction (XRD). a) θ-2θ X-ray diffraction spectrum of the 2LNO/4CMO superlattice. The 0th-order superlattice peak (SL_0) is obscured by the (002) $LaAlO_3$ substrate peak at 2θ = 48º. The anticipated number of SL thickness fringes is eight (P − 2 = 8) for a superlattice with ten periods (P = 10). b) θ-2θ spectrum for the 8LNO/4CMO superlattice. The 0th-order superlattice peak (SL_0) is detected at 2θ = 47º. Both superlattices exhibit prominent 1st-order superlattice peaks labeled as SL_{-1} and SL_{+1}.

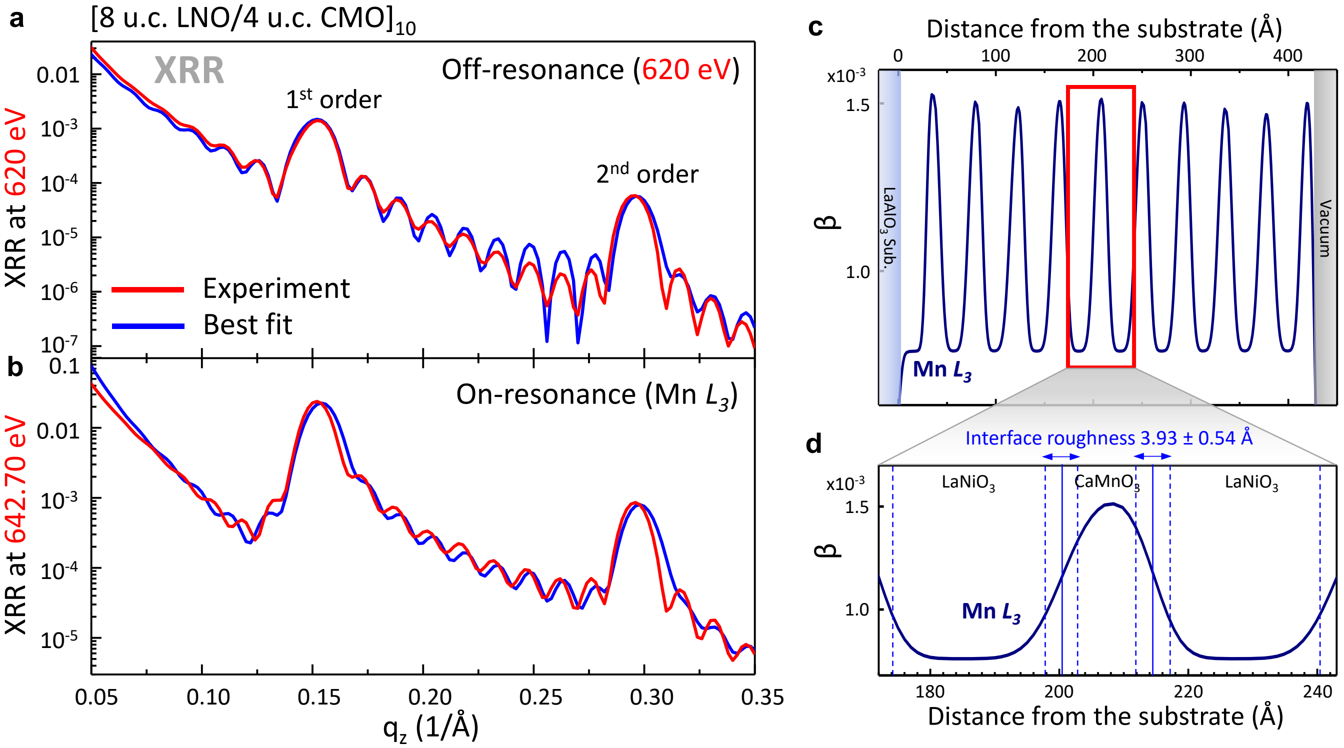

Figure S2. Structural Characterization via Soft X-ray Reflectivity (SXR). a) and b) Momentum-dependent SXR spectra (red curves) and the best fits (blue curves) to the experimental data measured at the photon energies corresponding to off-resonant (620 eV) and resonant (Mn L_3 at 642.7 eV) conditions, respectively. Self-consistent fitting of the data yields a detailed optical (absorption coefficient β) profile of the sample, shown in c) and d) with the resultant layer thicknesses of 28.46 ± 0.22 Å (~7.5 u.c. of $LaNiO_3$) and 14.25 ± 0.08 Å (~4 u.c. $CaMnO_3$), as well as the average interface roughness (chemical interdiffusion) of 3.93 ± 0.54 Å.

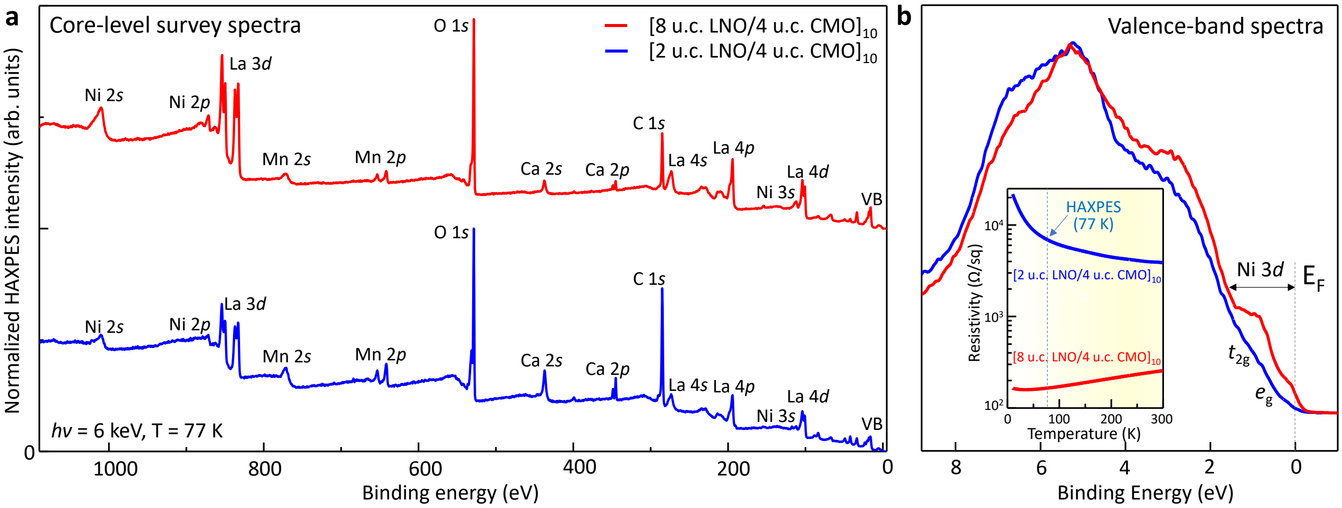

Figure S3. Chemical and Electronic Characterization. a) Bulk-sensitive HAXPES survey spectra for the 8LNO/4CMO (red line) and 2LNO/4CMO (blue line) superlattices. The presence of all expected elements (Ca, Mn, O, La, Ni, and C from the surface-adsorbed contaminant C/O layer) is confirmed by corresponding core-level peaks. b) Angle-integrated valence-band spectra of the same superlattices recorded with a photon energy of 6 keV. Considerable suppression of the near-E_F Ni 3*d* e_g and t_{2g} density of states results in a metal-insulator transition in the 2LNO/4CMO sample, as probed with sheet resistivity measurements shown in the inset.

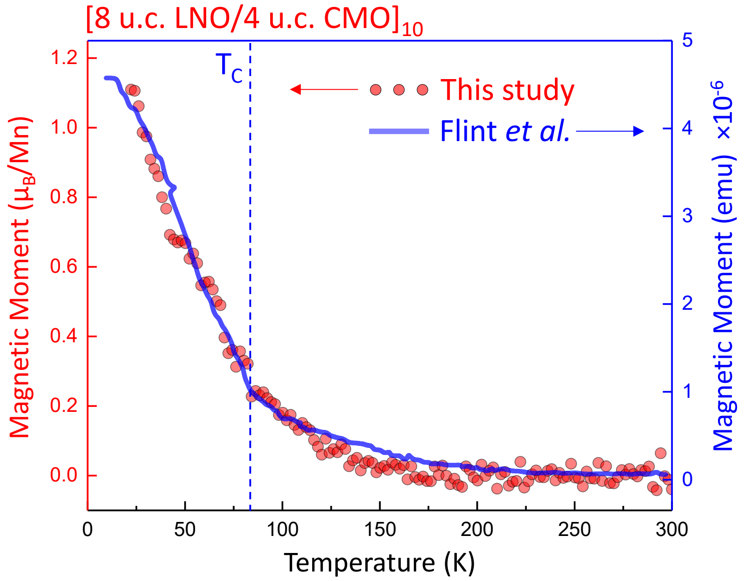

Figure S4. Magnetic Characterization. Temperature dependence of the Mn magnetic moment for the 8LNO/2CMO superlattice obtained in a warming field of 2 T after field-cooling in a 7 T field (red symbols, left y-scale). The standard background subtraction procedure described in Ref. [86] was utilized to isolate the superlattice contribution to the magnetic signal. Subsequently, the data were normalized to the estimated number of interfacial Mn ions in the superlattice to obtain the values of μ_B per interfacial Mn. The data are compared to the M vs. T curve obtained in a prior study [74] on a similar (6LNO/4CMO) superlattice (blue curve, right y-axis).

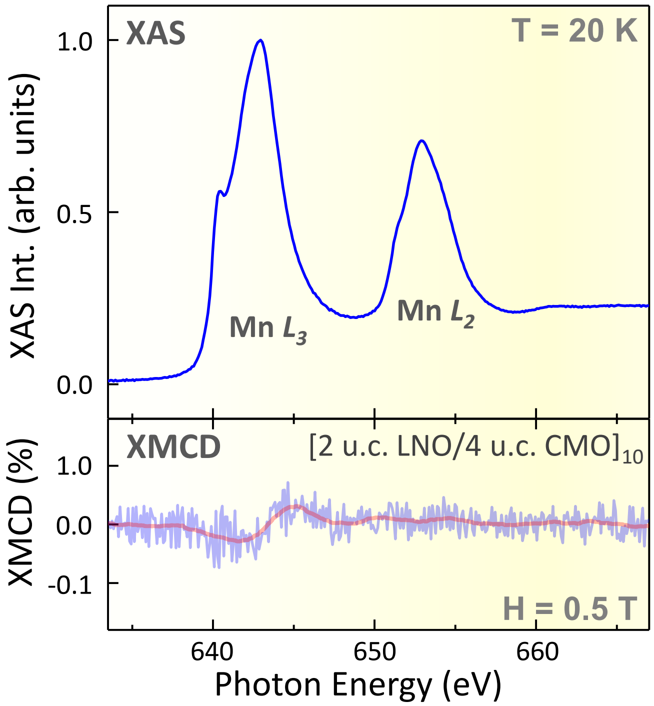

Figure S5. XAS/XMCD Measurements of the 2LNO/4CMO Reference Sample. Top panel: Bulk-sensitive Mn $L_{2,3}$-edge XAS spectrum, measured in luminescence detection mode at T = 20 K and probing the entire depth of the 2LNO/4CMO reference sample. Bottom panel: XMCD spectrum measured in an in-plane magnetic field of 0.5 T shows only a residual (considerably suppressed) magnetic signal compared to the 8LNO/4CMO sample (see Figure 1a in the main text for comparison). The light-blue spectrum represents the raw data, while the solid red curve, smoothed using the Savitzky-Golay method, serves as a guide to the eye.

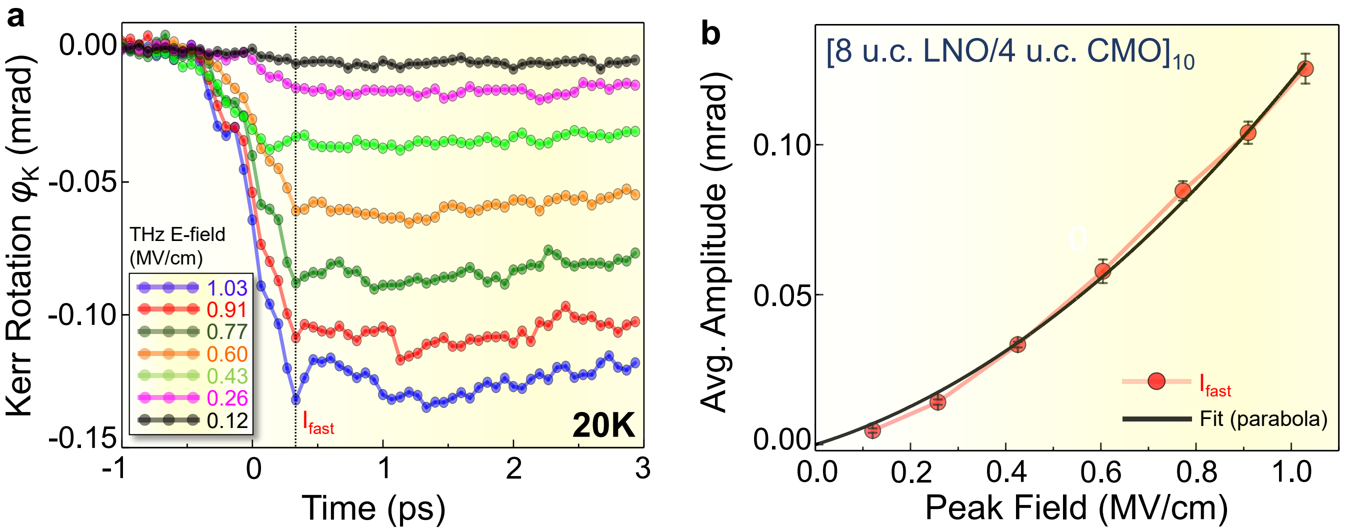

Figure S6. Fluence-dependent THz-pump tr-MOKE measurements. a) Fluence (THz peak E-field) dependent THz-pump tr-MOKE time-delay traces recorded on the 8LNO/2CMO superlattice at T = 20 K. b) Average amplitudes of the magnetic response (Kerr rotation angle ϕ_K) for the 'fast' electronic response (red symbols) and the best fit of the experimental data to a parabola (black solid line).

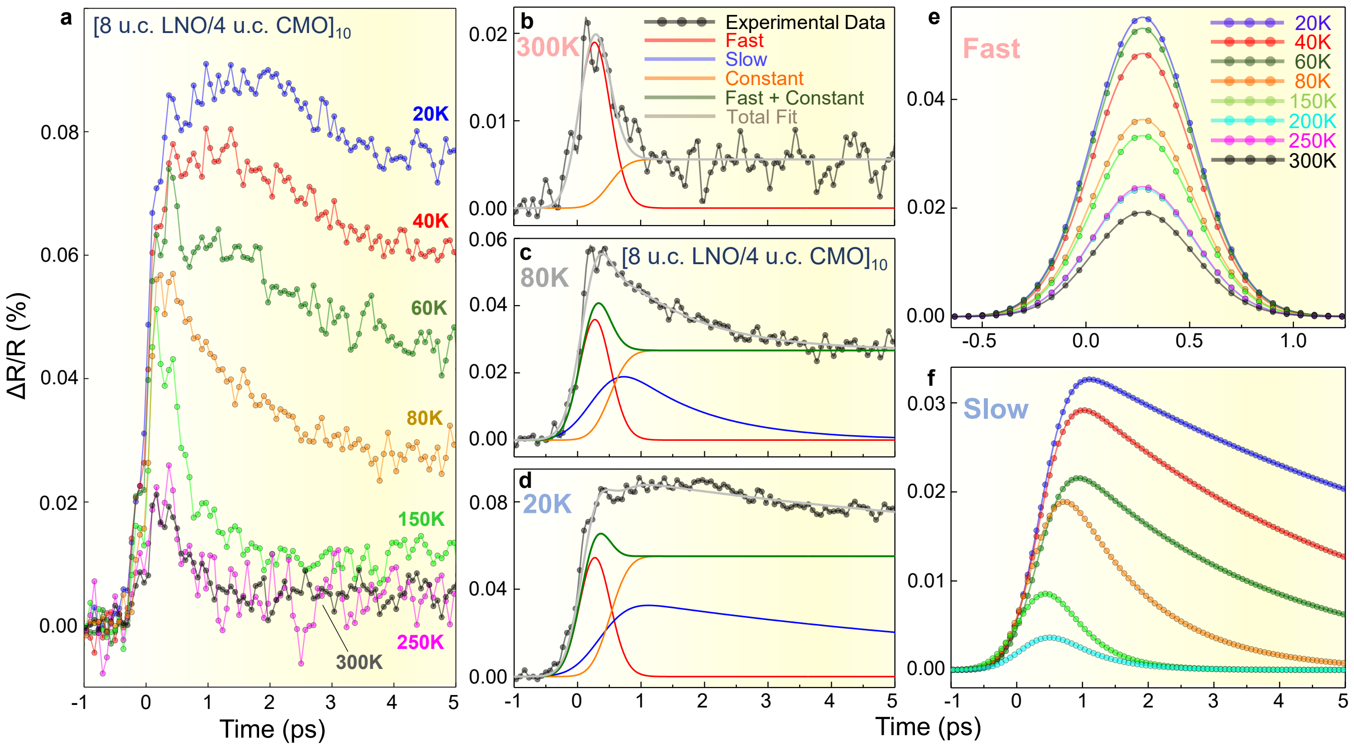

Figure S7. Ultrafast Temperature-Dependent THz-pump near-IR Reflectivity (ΔR/R) Measurements. a) Temperature-dependent ΔR/R delay traces recorded at several temperatures varying from 300 to 20 K. b) Temporal decomposition of the room-temperature (300 K) delay trace into the initial fast and quasiconstant components. c) Temporal decomposition of the delay trace recorded at T_C = 80 K requires an additional dynamic component characterized by a slower risetime and consequent exponential recovery (blue curve). d) Temporal decomposition of the 20 K delay trace requires all three dynamic components (fast, quasiconstant, and slow). e) All the temperature-dependent fits of the fast dynamic component, exhibiting uniform temporal behavior. f) Temperature-dependent fits of the slow dynamic component, which becomes prominent at the onset of interfacial ferromagnetism (T < 80 K).
